# Technology toolbox for cell line development - next generation cell line development technologies

**DOI:** 10.1186/1753-6561-9-S9-P17

**Published:** 2015-12-14

**Authors:** Holger Laux, Ursula Bodendorf, Sandrine Romand, Anett Ritter, Thomas Jostock, Burkhard Wilms

**Affiliations:** 1 Novartis Development Integrated Biologic Profiling, 4002 Basel, Switzerland; 2 Novartis Institutes for Biomedical Research, 4056 Basel, Switzerland

## Background

Chinese hamster ovary (CHO) cells are the most widely used host for large scale production of recombinant therapeutic proteins. A combination of several gene editing approaches applied to Novartis proprietary CHO cell line resulted in a superior cell line with a significant increase of titer and improved product quality. Inter alia we have surprisingly identified a key protease responsible for proteolytic degradation of mainly non-antibody format therapeutic proteins. The recently published CHO genome in combination with screening methods and cell line engineering tools has enabled the development of this novel CHO cell line.

## Materials and methods

CHO cell lines growing in suspension were cultivated at 36.5 C° and 10% CO2 in shake flasks using a proprietary, chemically defined culture medium. Gene editing was performed according to manufacturer's protocols, cells were single cell sorted using FACS device and subsequently screened for the desired phenotype. Cell viabilities and growth rates were monitored using an automated system (ViCell, Beckman Coulter). Cells were stably transfected by electroporation (Amaxa Nucleofection system, Lonza, Germany) with expression plasmids encoding human monoclonal antibodies. This and all other kits were used according to the manufacturer's instructions.

Spike-in experiments were performed in conditioned medium. Conditioned medium was collected by centrifugation of the cells grown for 7/8 days for 15 min at 90 g. After centrifugation, the supernatant was transferred and passed through a 0.22 μm filter to remove remaining cell particles from the conditioned medium. Under these conditions and at this stage of cell growth, the maximum amounts of secreted proteases are expected to be active in the cell culture medium without release of intracellular proteases due to cell death. The polypeptide of interest was added to the conditioned medium with a final concentration of 0.7 μM and incubated at 37°C with continuous shaking at 500 rpm. After incubation, samples were analyzed by SDS-PAGE followed by Western Blot analysis to determine the amount of clipping.

## Results

### Elimination of telomeric region of chromosome 8

**Figure 1:**
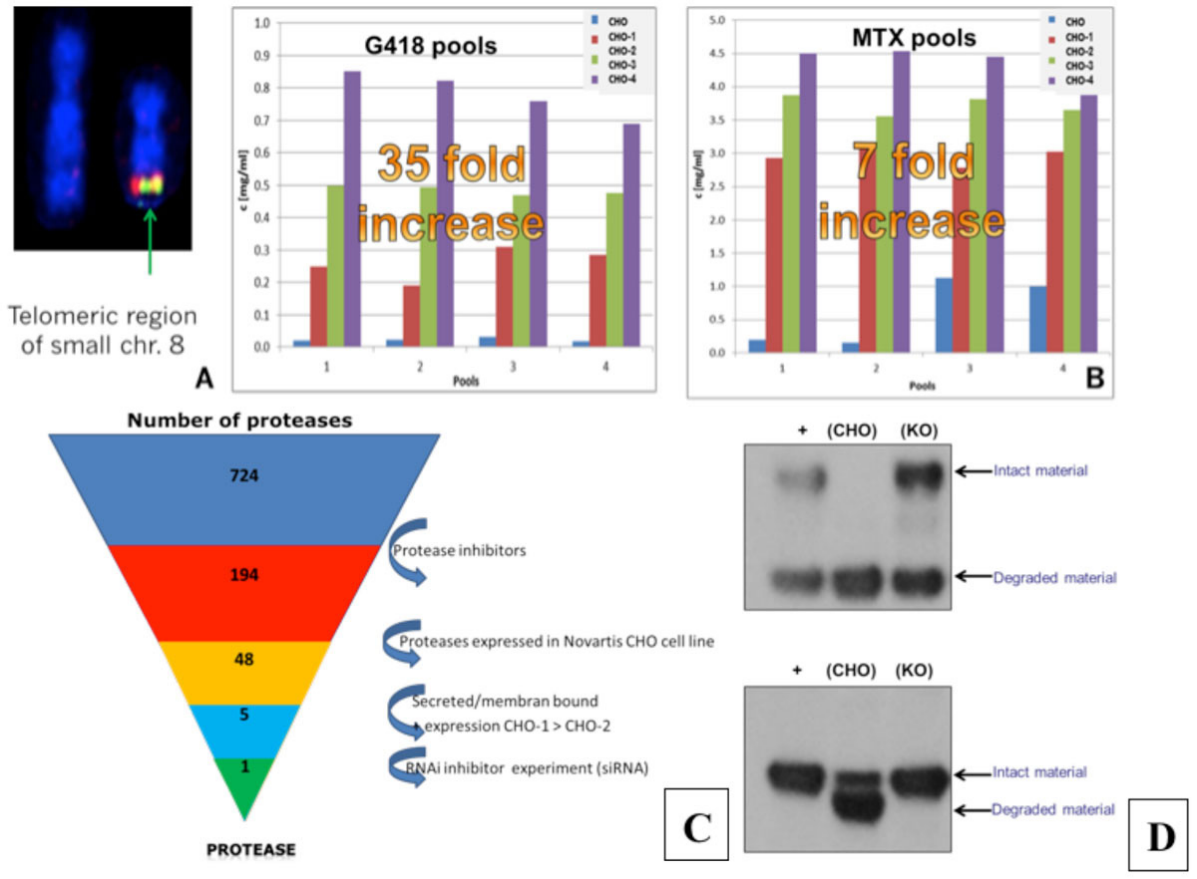
****A**: BAC hybridisation of relevant region to the large and small chromosome 8**. Only the small chromosome 8 contains the region of interest. **B: **Comparison of G418 batch and MTX fed-batch titers expressed in the three new CHO cell lines in comparison to CHO-K1 cells. A massive increase of pool productivity could be detected. **C: **A variety of assays were applied to identify the key protease. These include broad spectrum protease inhibitors, mass spectrum analysis, next generation sequencing and siRNAs. **D: **Evaluating proteolytic degradation in conditioned medium between WT cell line and protease KO cell line

Gene expression profiles of high versus low producing CHO clones were compared to identify genes correlated with increased titer. Improved production rates were surprisingly correlating with loss of the telomeric region of chromosome 8 (figure 1A). Based on this finding three new parental CHO cell lines lacking this region were generated and their capability for protein production was assessed. After transfection and two consecutive selection steps (geneticin followed by methotrexate selection) a massive increase of productivity could be detected for all three new cell lines compared to the original cell line (35 fold increase for G418 selection and 7 fold increase for methotrexate selection, respectively) (figure 1B). The increased productivity obtained with the new cell lines is facilitating the supply of early drug substance material. In addition, significantly more cell clones with a higher average productivity were obtained after single cell cloning. This results in reduced efforts in single cell sorting and the need to screen fewer clones.

### Identification of major clipping protease and gene knockout using gene editing technologies

Proteolytic activity in cell cultures is derived from endogenously expressed proteases. However of the major challenge is that more than 700 endogenous proteases are known. We applied different approaches e.g. a variety of protease inhibitors, siRNAs and next generation sequencing approaches to identify the main protease(s) involved in clipping of therapeutic proteins produced in CHOK1 derived cell lines (figure 1C).We have surprisingly identified one protease which is mainly responsible for proteolytic degradation. To eliminate the proteolytic activity, the protease gene was knocked out using gene editing technology. To evaluate the effect of the knockout cell line, protein candidates of diverse formats which were degraded in CHO wildtype cells were co-incubated in conditioned medium derived from knockout and wildtype CHO cell lines. No or significant reduced degradation of the proteins was detected using the protease knockout cell line (figure 1D).

## Conclusions

The combination of the recently published CHO genome [1-3]with screening methods and cell line engineering tools has enabled the development of a superior CHO cell line. Gene expression profile analysis of high vs. low antibody producing CHO cell lines revealed that high production is correlated with loss of the telomeric region of chromosome 8. New parental cell lines lacking this telomeric region showed increased productivities after transfection. Additionally the knockout of a surprisingly identified protease eliminates/significantly reduces proteolytic degradation evaluated with a variety of therapeutic proteins. In conclusion novel cell line engineering methods are powerful tools to improve productivity and can solve issues in production of therapeutic proteins in biopharmaceutical industry in a short timeframe with minimal screening effort.
